# CRISPRcruncher: A tool for engineering restriction sites into coding regions

**DOI:** 10.17912/micropub.biology.000343

**Published:** 2021-01-18

**Authors:** Samuel F. Fay, David S. Fay, Vikram E. Chhatre

**Affiliations:** 1 Wyoming INBRE Bioinformatics Core; 2 Department of Molecular Biology, University of Wyoming, Laramie, WY USA

## Abstract

CRISPR/Cas9 genome editing strategies often rely on the placement of an introduced restriction endonuclease (RE) site adjacent to the genomic edit of interest. This allows for rapid initial PCR-based detection of cells and organisms containing the edit of interest and may also be used for subsequent genotyping. Nevertheless, engineering RE sites at optimal locations within coding regions can be difficult due to the many hundreds of potential endonuclease options and the strict requirement to maintain the correct amino acid sequence. Here we report CRISPRcruncher, a computational tool that analyzes an input coding sequence and produces a complete list of all possible changes that could be made that will create new RE sites while preserving the original peptide sequence. Notably, for sequences tested, CRISPRcruncher identified approximately one new RE site per input nucleotide when mining for 4-bp or longer RE motifs and 0.5 new RE sites per input nucleotide when mining for 6-bp or longer motifs. Therefore, CRISPRcruncher represents a powerful new computational tool in the CRISPR arsenal.

**Figure 1. Summary of CRISPRcruncher:**
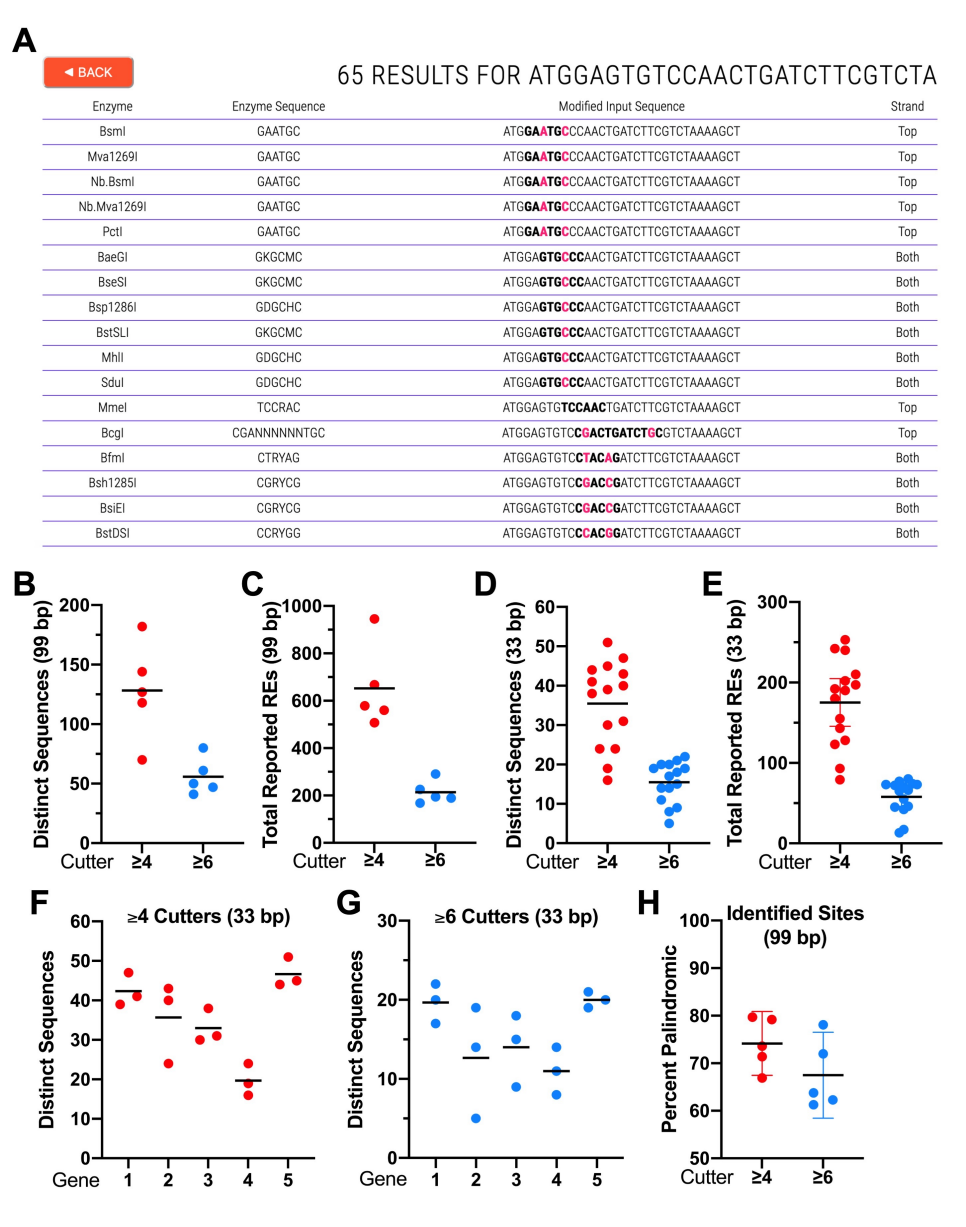
(A) Example CRISPRcruncher output displaying options for introducing restriction sites (≥6 bp) within the first 33 coding nucleotides of CELE_F54B11.2. For simplicity, only 17/65 lines of output are shown. Red letters indicate changes from the input sequence; bold letters indicate the newly introduced endonuclease recognition site. “Strand” column indicates whether the top (shown) or bottom strand contains the recognition motif; both indicates that the motif is an inverted repeat (a.k.a. palindrome). (B–G) Analyses of site frequencies for five randomly selected genes on the X chromosome. The first 99 coding nucleotides within the first exon of each gene were examined either as a contiguous sequence (B,C) or as three nonoverlapping 33-bp segments (D–G). “Total Reported REs” indicates the complete set of REs, including isoschizomers and other categories of related motifs, capable of cleaving at the identified new sites. “Distinct Sequences” indicates the total number of distinct new 33-bp or 99-bp sequences identified by CRISPRcruncher. Parameters were set to identify new restriction sites of 4 bp or greater (≥4 cutter; red circles) or 6 bp or greater (≥6 cutter; blue circles). (H) Percent of palindromic versus non-palindromic restriction motifs for the five 99-bp sequences. For supporting information, see Extended Data.

## Description

The precise engineering of genomes using CRISPR/Cas9 homology-directed repair has become largely routine, particularly for making small changes such as nucleotide substitutions (Dickinson and Goldstein, 2016; Farboud, 2017; Sternberg and Doudna, 2015). This approach is often accompanied by the introduction of a restriction endonuclease (RE) site positioned adjacent to the modified target region (Friedland *et al.*, 2013; Lo *et al.*, 2013; Paix *et al.*, 2015; Waaijers *et al.*, 2013; Zhao *et al.*, 2014). Such RE sites allow for rapid PCR-based screening to detect cells or organisms containing the desired edit and greatly aid in subsequent genotyping. In addition, the precise location of the engineered RE site relative to the protospacer adjacent motif (PAM) site and the region targeted for modification can impact the efficiency of CRISPR (Farboud *et al.*, 2019). Currently, most labs design these introduced RE sites without the use of a computational tool (i.e., ‘by hand’), which is inefficient and likely to overlook a large majority of engineerable sites.

To facilitate and improve CRISPR-repair design, we developed CRISPRcruncher (https://crisprcruncher.io), a web-based computational tool written in Python (Van Rossum 2007) that scans DNA coding regions from 15 to 99 bp in length and reports a complete list of minimal changes that result in introduced RE sites. Importantly, changes identified by the tool always maintain the original encoded amino acid sequence. In brief, the tool exploits codon degeneracy along with a built-in library of ~650 RE consensus motifs (Extended Data). CRISPRcruncher scans coding regions in a 5’ to 3’ direction using a series of 15-bp sliding windows, each offset by 3 bp (the reading frame). The end user, in addition to entering the DNA sequence, can also choose a minimum cutoff length for the reported RE sites (e.g., ≥6 bp). We define ≥6 bp cutters as REs that recognize motifs containing six or more bp as part of their core recognition sequence. As such, both HindIII (5’-AAGCTT-3’) and the 8-bp cutter, NotI (5’-GCGGCCGC-3’), are ≥6 bp cutters. Likewise ≥4 bp cutters include 4-bp cutters, such as AluI (5’-AGCT-3’), as well as REs with longer recognition motifs, such as HindIII and NotI (also see discussion below). The output lists the identified REs together with their corresponding sites in a 5’ to 3’ order (Figure 1A). The output indicates the altered nucleotides and strand location (top, bottom, or both) and places isoschizomers, distinct enzymes with identical recognition motifs, on separate lines (Figure 1A).

To get a sense for how many potential new RE sites can be identified by CRISPRcruncher, we examined the first 99 coding nucleotides within the first exon of five randomly chosen genes on the X chromosome of *Caenorhabditis elegans* (Extended Data). In searches for 4-bp or longer RE recognition sites (≥4 cutters), we obtained an average of 128 distinct 99-bp sequences (range, 70–182), that correspond to an average total of 652 total reported REs (range, 508–945) (Figure 1B,C; Extended Data). In searches for 6-bp or longer RE recognition sites (≥6 cutters), we obtained an average of 56 distinct 99-bp sequences (range, 41–80), that correspond to an average total of 213 total reported REs (range, 168–291) (Figure 1B,C; Extended Data). The difference between the numbers obtained for ‘distinct sequences’ and ‘total reported REs’ is due in part to isoschizomers and also to enzymes with related but divergent consensus motifs that can recognize the same distinctly altered sequence (Extended Data). For example, by changing 5’-CCTGGG-3’, which encodes Pro-Gly, to 5’-CCCGGG-3’, which also encodes Pro-Gly, sites for XmaI (5’-CCCGGG-3’), BsoBI (5’-CYCGRG-3’), and HpaII (5’-CCGG-3’) are simultaneously created.

As expected, using a 6-bp cutoff for recognition motifs (≥6 cutters) we observed fewer ‘distinct sequences’ and ‘total reported REs’ than when using a ≥4-bp cutoff (Figure 1B,C; Extended Data). Notably, these data indicate that on average more than one distinct sequence can be identified per 2 bp of coding sequence for ≥6 cutters and more than one distinct sequence can be identified per each base pair for ≥4 cutters. With respect to the availability of enzymes, a quick survey of ≥6 cutter sites identified within the first 33 bp of CELE_ F54B11.2 indicated that a very high proportion (21/21) can be cleaved using enzymes that are commercially available through at least one major vendor in the United States (Extended Data). Thus, the RE sites identified by CRISPRcruncher are both abundant and useful.

We also examined distinct sequences and total reported enzymes for 15 non-overlapping 33-bp DNA segments derived from the five 99-bp regions (Figure 1D,E). For ≥4 cutters we observed averages of 36 ‘distinct sequences’ (range, 16–51) and 175 ‘total reported REs’ (range, 79–253), whereas for 6 cutters we observed averages of 16 ‘distinct sequences’ (range, 5–22) and 58 ‘total reported REs’ (range, 13–80). A further breakdown of these parameters for each of the five genes is shown in Figure 1F,G. These numbers are consistent with those reported for the 99-bp segments but are slightly lower due to the absence of RE sites spanning the sequence breaks at 33 bp and 66 bp. Overall, CRISPRcruncher identified about one distinct change per 2 bp for ≥6 cutters and one distinct change per each base pair for ≥4 cutters. Nevertheless, as can be seen (Figure 1F,G; Extended Data), the distribution of RE sites can vary considerably between regions due to factors including DNA sequence composition and codon flexibility.

Several additional points are worth noting. (1) CRISPRcruncher ‘counts’ degenerate base symbols, such as R (A or G) and Y (C or T), the same as it does G, A, T, and C. Thus, BanII (5’-GRGCYC-3’) and BaeGI (5’-GKGCMC-3’) are both considered 6-bp cutters, even though their genomic site frequencies will be greater than that of ApaI (5’-GGGCCC-3’). In contrast, the non-specific designation (N) is not factored into motif size. Thus, NlaIV (5’-GGNNCC-3’) is considered a 4-bp cutter. (2) Because of codon redundancy and RE motif degeneracy, there will be cases when multiple different substitutions at the same nucleotide position can produce a consensus match for a given RE while preserving the original amino acid sequence. For example, changing 5’-ACCTGG-3’, which encodes Thr-Trp, to either 5’-ACATGG-3’ or 5’-ACGTGG-3’ will preserve the amino acid sequence, and both substitutions will create a consensus site for AflIII (5’-ACRYGT-3’). CRISPRcruncher will report only one of these changes; however, it may be advisable to consider both options if, for example, codon bias is an issue. This can easily be done by checking codon usage tables along with the relevant RE consensus motif to see if the ‘non-reported’ alternative nucleotide is preferrable. (3) Some changes may be made outside RE consensus motifs. For example, the sequence 5’-GATCTTCGT-3’, which encodes Asp-Leu-Arg, can be changed to 5’-GACTTAAGA-3’, creating a new site for AflII (5’-CTTAAG-3’) while preserving the amino acid sequence. In this case, the 3’-most substitution occurs outside the consensus motif for AflII (i.e., both CGT and AGA encode Arg). As can be appreciated, this type of RE site creation would be difficult to accomplish without the use of a computational tool. (4) Stop codons are treated identically to amino acids. Thus, 5’-TAA-3’ may be changed to either 5’-TAG-3’ or 5’-TGA-3’. (5) An analysis of RE sites identified by CRISPRcruncher indicates that whereas ~70% are palindromic (a.k.a., inverted repeats), ~30% are not (Figure 1H). Because non-palindromic motifs are generally more difficult to design without computational tools, this subclass represents an additional benefit of using CRISPRcruncher.

We also note that CRISPRcruncher is not designed to take into account codon bias, guide-RNA preferences, or other relevant repair-template design considerations, all of which will be important in achieving success with CRISPR editing. Many other tools and resources address these concerns and can be easily used in conjunction with CRISPRcruncher. In addition, although CRISPRcruncher reports RE sites that are already present within the input sequence, it does not make suggestions for ablating these sites. In most cases, such sites will either be too abundant for screening purposes (e.g., many 4-cutters) or will be located at non-optimal positions when taking into account repair design principles. In rare cases where they may prove useful, disruption of such RE sites is easily accomplished ‘by hand’.

In summary, CRISPRcruncher is a powerful new design tool for introducing useful RE sites into repair templates. CRISPRcruncher should be used in combination with (i) current tools for predicting the efficiency and specificity of guide-RNAs, (ii) species-specific codon usage tables, and (iii) validated repair-template design principles. In some cases, choice of the engineered RE site may come down to which enzymes are already available to the lab, thereby reducing costs.

## Methods

CRISPRcruncher is available as a web-based tool at https://crisprcruncher.io. The Python source code (release v1.0) is available on CaltechData (10.22002/D1.1861). Future updates to the code will be maintained at https://github.com/samuelfay/CRISPRcruncher.git. The standalone script may be implemented locally on Windows, Mac OSX and Unix platforms to achieve greater computational efficiency. A user manual and interpretation guide are available.

Extended data are archived and available on Caltech Data (10.22002/D1.1862).

Information regarding RE motifs and other properties was obtained primarily from (https://www.neb.com). Analyses associated with Figure 1 were done in GraphPad Prism 9.

